# Editorial: Stem cells in oral cavity: from development to regeneration, Volume II

**DOI:** 10.3389/fcell.2023.1207744

**Published:** 2023-04-28

**Authors:** Takehito Ouchi, Giovanna Orsini, Mikihito Kajiya, Anne George

**Affiliations:** ^1^ Department of Physiology, Tokyo Dental College, Tokyo, Japan; ^2^ Department of Clinical Sciences and Stomatology, Università Politecnica delle Marche, Ancona, Italy; ^3^ Department of Innovation and Precision Dentistry, Hiroshima University Hospital, Hiroshima, Japan; ^4^ Brodie Tooth Development Genetics and Regenerative Medicine Research Laboratory, College of Dentistry, University of Illinois at Chicago, Chicago, IL, United States

**Keywords:** development, genetics, skeletal stem cells, mesenchymal stem cell, regeneration, oral cavity

Oral cavity is a unique place, as the first digestion system and also close to the brain, which is essential conductor in vertebrates. The oral function is precisely maintained by both soft and hard tissues, and totally orchestrated by the central nerve system. In the previous edition, which is entitled “Stem Cells in Oral Cavity: From Development to Regeneration”, we learned several kinds of oral cell type potentials in biological functions and regenerative medicines (Ouchi et al., 2022). However, at the same time, we realized that further studies through multifaceted evaluations, by combining functional analysis under physiological and pathological conditions, will be needed and will definitely strengthen this research field toward clinical outcomes for patients and next-generation therapies. In the second edit, several studies were discussed, and researchers provided additional hope of potentials of oral and cranial stem cells in their application. Zhang et al. and his group demonstrated that dental pulp stem cells (DPSCs) can differentiate into vascular endothelial cells that form functional blood vessels that mature upon recruitment of host smooth muscle cells (Zhang et al.). The process of DPSC-derived vessel maturation is regulated by platelet-derived growth factor-BB (PDGF-BB) signaling through PDGFR-β. This is a critical cellular event that is required for the stabilization and long-term function of blood vessels. DPSCs are reported to proliferate and are capable of generating hard tissues (Gronthos et al., 2000). Zhang et al.’s work provides insights that can be used for the development of mechanism-based therapies for tissue regeneration that involve vasculogenic differentiation of mesenchymal stem cells (MSCs). As new aspects, the cell plasticity or unexpected lineage change will be one of routes in maintaining the biological functions (Mattioli-Belmonte et al., 2015).

Cellular features are determined by the effect of specific protein expression or transcription factors. The RNA and protein expression patterns are gradually changing as cellular fate changes. Single cell analysis provides the information to study cell-to-cell interactions and variations within a cell population in the vertebrate organ. A comprehensive and integrated bird’s-eye view of the transcriptome landscape of human and mouse teeth is now a powerful multifaceted tool for dental research, which will further decipher dental biology and continue to advance this field. Hermans et al. used recently published single cell RNA-sequencing datasets of different human and mouse tooth tissue types (healthy and diseased teeth) to establish a comprehensive dental single cell atlas. Data were re-evaluated and merged, and the power of applying the merged data map was explored. Authors identified a novel candidate transcriptional regulator of the ameloblast lineage in mouse teeth. As for human teeth, it supports previously undeveloped developmental connections between specific epithelial compartments. Authors have established a comprehensive murine and human dental single cell atlas as a powerful tool for enhancing innovative research in dental biology, development, and disease (Hermans et al.). Thus, single cell RNA-sequencing based on several ages and multiple different vertebrate species can further construct non-biased single cell level gene features in each type of oral and dental cells.

Genetic regulation and mutations impact the oral and dental physiological functions such as breathing, eating, talking, and swallowing. And these functions are regulated by the brain, local environmental niche, and also the temporomandibular joint (TMJ), which is essential part connecting the cranial base and mandibular jaw. In other words, organs in the craniofacial and oral cavity are precisely controlled by higher brain function through the neurons and play essential roles in several life processes. TMJ also supports dental and oral function as an important intermediate structure. Recent lineage tracing technology can provide visualization of the genes *in vitro* and *in vivo*. Gli1 is a marker for MSCs and progenitors in the craniofacial bones, incisors, and periodontal ligaments of adult mice (Zhao et al., 2014; Zhao et al., 2015; Men et al., 2020). However, the potential relationship between Gli1 and postnatal skeletal progenitors in the condyle has not been investigated. Gli1 functions as the read-out of endogenous hedgehog (Hh) signaling activity and a transcription factor that regulates the expression of Hh target genes. Chen et al., sorted the Gli1^+^ cells from condylar subchondral bones and cultured them *in vitro* (Chen et al.). The cells exhibited self-renewal, colony-forming, and osteogenic differentiation capacities. However, proliferation and differentiation were significantly downregulated when Wnt signaling was inhibited. Thus, Wnt/β-catenin signaling may mediate the osteogenesis potential of Gli1^+^ cells in the condyle. In summary, authors have uncovered a population of osteogenic progenitors that could be labeled with Gli1 in the mandibular condyle. These Gli1^+^ cells reside at the chondro-osseous junction immediately beneath the cartilage. Gli1^+^ progenitors contribute to the osteoblast lineage during postnatal development, homeostasis, and fracture repair of the condyle. Wnt/β-catenin signaling may be a crucial driving force for the osteogenic differentiation of Gli1^+^ MSCs. The study provided a potential target that could promote condylar growth and fracture healing.

The expression profiles of exosomal microRNAs (miRNAs) are regulated by the microenvironment. Recent studies have reported the therapeutic potential of the MSCs-derived secretome, which includes trophic factors such as cytokines, growth factors, and extracellular vesicles (EVs) (Pethő et al., 2018). Nishimura and colleagues identified miR-1260b as a novel TNF inducible gingival MSC-derived exosomal miRNA (Nakao et al., 2021). miR-1260b was reported to be downregulated in gingival tissue from periodontitis (Stoecklin-Wasmer et al., 2012). Hayashi et al., reported that activating transcription factor 6β, which is one of endoplasmic reticulum (ER) stress relating protein (Hien and Back, 2021), but still poorly understood, is the top-ranked target of miR-1260b in multiple miRNA target database analysis in the mice periodontal model. miR-1260b-mediated regulation of ER stress may be a potential target for the inhibition of alveolar bone loss (Hayashi et al.). Organelles of the cells in the oral tissue is an essential component to maintain physiological functions. Impairment of the organelles can induce functional disability.

Homeostasis maintained by the feedback loop from intra and extracellular regulation to biological function and *vice versa* is always an essential mechanism in oral tissues. Understanding the formation of oral tissues and the involvement of the brain in this process will further clarify the oral stem cell behaviors, developmental fates, determination fate, and subsequent oral physiological functions.
